# Microplastics identified in commercial over-the-counter lubricant eyedrops

**DOI:** 10.1038/s41433-025-04031-6

**Published:** 2025-09-29

**Authors:** Julia Jaeger, Matthew Burke, Duoduo Wu, Emily Ern-Min Curren, Sandric Chee Yew Leong, Robert Symons, Blanche Xiao Hong Lim, Xinyi Su, Jodhbir Singh Mehta, Andri Kartasasmita Riau, Chris Hong Long Lim

**Affiliations:** 1 Eurofins Environment Testing Australia & New Zealand, Dandenong South, VIC Australia; 2 Department of Ophthalmology, National University Health System, Singapore, Singapore; 3 St. John’s Island National Marine Laboratory, Tropical Marine Science Institute, National University of Singapore, Singapore, Singapore; 4 Eurofins Environment Testing Australia & New Zealand, Girraween, NSW Australia; 5 Centre for Sustainable Medicine, Yong Loo Lin School of Medicine, National University of Singapore, Singapore, Singapore; 6 Singapore Eye Research Institute, Singapore, Singapore; 7 Ophthalmology and Visual Sciences Academic Clinical Programme, Duke-NUS Medical School, Singapore, Singapore; 8 Singapore National Eye Centre, Singapore, Singapore; 9 Cornea and Oculoplastics Units, Department of Ophthalmology, Royal Perth Hospital, Perth, WA Australia

**Keywords:** Medical research, Eye diseases

## Abstract

**Background:**

There is increasing evidence that microplastics exert harmful effects on human health and on the ocular surface. This pilot study aims to investigate if microplastic particles are present in commonly used eyedrops in single-use plastic vials.

**Methods:**

Six commonly used commercial tear-replacement solutions available without a doctor’s prescription were tested (Brands A-F). All brands of eyedrops were analysed visually using light microscopy and the number of microplastic particles were manually counted. These were further analysed using the Agilent 8700 Laser Direct Infrared (LDIR) chemical imaging system.

**Results:**

All eyedrops analysed contained microplastics. The number of particles identified using light microscopy ranged between 15 (Brand E) to >18,000 (Brand F). In total, nine types of microplastics were identified with LDIR – polyethylene, polypropylene, polystyrene, polyvinylchloride, polyethylene terephthalate, polycarbonate, polymethylmethacrylate, polyamide and polyurethane.

**Conclusions:**

This study provides the most extensive quantification of microplastic contamination in commercial eyedrops to date, highlighting a major, under-recognised source of ocular exposure. These particles may arise from secondary degradation of plastic vials during production, transportation, storage, or during instillation, or introduced from other sources during production and represent a major source of exposure to the ocular surface, especially among patients who require chronic instillation of eyedrops.

## Introduction

Plastics are synthetic or semi-synthetic polymeric materials found in everyday items. Their properties allow them to be moulded into objects with varied shapes and rigidity, making them invaluable in daily life and especially so in healthcare [1]. However, plastics have been shown to undergo secondary degradation into smaller pieces, termed microplastics and nanoplastics.

Microplastics are defined as plastic particles smaller than 5 mm [2]. Primary microplastics are manufactured plastic particles of small sizes, while secondary microplastics are derived from physical, chemical, and biological degradation of larger plastic materials found in the environment. Their presence is ubiquitous and microplastics have been isolated from air, water, food, and humans [3, 4]. Although the level of microplastic exposure differs depending on environmental and lifestyle factors, it is estimated that children and adults ingest 553 and 583 microplastic particles per day respectively [5, 6].

There is a growing body of evidence that microplastic exposure may be associated with significant health risks. Exposure has been implicated in conditions ranging from malignancies to endocrine and foetal developmental abnormalities [4, 7–9]. This threat extends to the ocular surface, which is exposed to microplastics from multiple sources. General environmental exposure occurs through atmospheric particulate matter, which population-based studies suggest may contribute to dry eye disease [10–12]. Direct ophthalmic sources are also significant; the healthcare sector's reliance on plastics leads to microplastic contamination in surgical environments [13], and contact lenses alone can release an estimated 90,000 particles onto the ocular surface annually [14]. This exposure is not benign. In murine models, exposure of the ocular surface to microplastics has been associated with upregulation of inflammatory cytokines [15]. These experimental findings are mirrored in human clinical studies, where microplastics have been isolated from vitreous samples of patients undergoing intraocular surgery, with higher concentrations linked to elevated intraocular pressure and aqueous humour opacities [16].

Given the ubiquitous nature of microplastics in plastic packaging, it is conceivable that microplastics may also be present in commercially available eyedrops. A recent study demonstrated the presence of microplastic particles in commercial artificial tears using Raman spectroscopy and scanning electron microscopy [17]. This is of particular concern in patients with chronic ophthalmic conditions such as glaucoma, dry eye disease or uveitides, requiring long-term instillation of eyedrops which may expose the ocular surface to microplastics. Thus far, there is limited published literature supporting the identification of microplastics in topical ophthalmic formulations. This study aims to identify and characterise microplastics in common over-the-counter tear-replacement solutions using light microscopy and laser direct infrared (LDIR) imaging.

## Methodology

### Sample collection

**Fig. 1:**
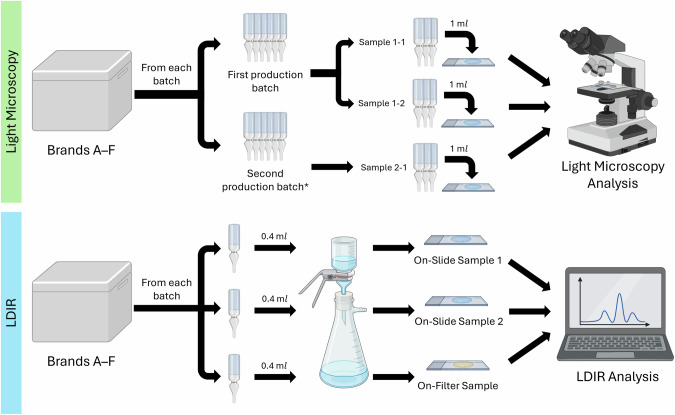
Summary experimental workflow of analysis with light microscopy and laser direct infrared imaging system with spectroscopy. *Samples were obtained from a second production batch whenever possible.

Single-use tear-replacement solutions of various brands and formulations were selected for this study. These over-the-counter commercial eyedrops with their constituent ingredients and volumes are summarised in Table 1. All eyedrop samples were analysed prior to their expiry date. Two methods of analyses – light microscopy with manual counting and LDIR imaging with an automated counting system were performed in a microplastics-free environment (Fig. 1). Details of methods undertaken are outlined below.

**Table 1:** Composition of selected tear-replacement solutions.

Brand	Ingredients	Volume
A	Carmellose sodium	0.4 ml
	Sodium chloride	
	Sodium lactate	
	Potassium chloride	
	Calcium chloride dihydrate	
	Magnesium chloride hexahydrate	
	Purified water	
	Sodium hydroxide	
	Hydrochloric acid	
B	Carboxymethylcellulose sodium	0.4 ml
	Sodium chloride	
	Sodium lactate	
	Potassium chloride	
	Calcium chloride dihydrate	
	Magnesium chloride hexahydrate	
	Purified water	
	Sodium hydroxide	
	Hydrochloric acid	
C	Carboxymethylcellulose sodium	0.4 ml
	Glycerin	
	Boric acid	
	Calcium chloride dihydrate	
	Erythritol	
	Levocarnitine	
	Magnesium chloride hexahydrate	
	Potassium chloride	
	Purified water	
	Stabilised oxychloro complex	
	Sodium borate decahydrate	
	Sodium citrate dihydrate	
D	Polyvinyl alcohol	0.4 ml
	Povidone	
	Sodium chloride	
	Purified water	
	Sodium hydroxide	
	Hydrochloric acid	
E	Dextran	0.8 ml
	Hypromellose	
	Potassium chloride	
	Purified water	
	Sodium borate	
	Sodium chloride	
	Sodium hydroxide	
	Hydrochloric acid	
F	Carboxymethylcellulose sodium	0.4 ml
	Glycerin	
	Polysorbate	
	Boric acid	
	Carbomer copolymer type A	
	Castor oil	
	Erythritol	
	Levocarnitine	
	Purified water	
	Sodium hydroxide	
	Hydrochloric acid

### Light microscopy

Glass and stainless steel labware were used to minimise external microplastic contamination. All labware (including forceps, glass bottles, petri dishes and metal sieves) were sterilised with ethanol and rinsed with Mili-Q ultrapure water twice prior to commencing sample processing and analysis. All open-lid containers were covered with either a glass slide or aluminium foil to minimise microplastics contamination from the environment. Loading of eyedrop samples was conducted in a clean air cabinet. A thorough description of contamination protocols undertaken during analysis were as described by Curren and Leong [18].

A total of three samples of each over-the-counter tear-replacement solution were tested. Two samples were from the same production batch while a third sample was obtained from another batch, when possible. This allowed for comparison of particle counts between samples of the same batch and between batches for each formulation. Samples from tear-replacement solutions were analysed by pipetting 1 mL of each eyedrop formulation using a glass pipette onto a Sedgewick rafter slide and viewed under an inverted light microscope. Microplastic particles were identified based on visual characteristics and sorted into categories such as fibre, film, or fragment. The number of particles in each sample was determined from triplicate counts. Blank controls were used to ensure that no ambient microplastic contamination was present prior to testing.

### Laser direct infrared imaging system with spectroscopy

LDIR is an infrared spectrometer that utilises a fast-tuneable quantum-cascade laser (QCL) coupled with a rapidly scanning imaging system to analyse particulate information such as quantification, size, colour and morphology, while identification of polymer type is identified via spectroscopic analysis [19].

To prevent environmental contamination, LDIR analysis was performed in a segregated room within the facility, physically distinct and equipped with an airlock system and change pod. The laboratory was kept at positive pressure with high efficiency particulate air (HEPA) filtration applied at the air inlet. Monthly air blank tests are conducted to ensure air quality within the laboratory and fume cupboard. Nightly vacuuming and regular surface wiping is also performed to remove potential contaminants.

Lab analysts donned specialised black cotton lab coats specially tailored to minimise polymer fibre shedding. All glassware underwent either furnace cleaning for a minimum of 4 h at temperatures exceeding 400 °C or washing with microplastics-analysis-grade (MAG) water. Prior to use, MAG water samples were analysed for purity and served as blank controls thereafter. Sample filtration was performed within a fume cupboard, with samples covered with foil.

Quality control and accuracy of analysis was ensured by running samples with a known concentration of green polyethylene and analysing reagent blanks using 1 L of MAG water. A regularly updated polymer register is also used to track and trace potential contaminations.

Reference materials for the analysis and spectral library were obtained from various manufacturers: fluorescent green polyethylene microspheres of 75–90 µm and 250–300 µm (Cospheric, Santa Barbara, CA, USA), polypropylene chromatographic grade of 150 µm (Polysciences Inc, Warrington, PA, USA), polystyrene EZY-CALTM microsphere size standard, NIST-traceable with mean diameter 30 or 70 µm, and a concentration of 2000 particles/mL (ThermoFisher Scientific Waltham, MA, USA), polyvinylchloride analytical standard (Sigma-Aldrich, Saint Louis, MO, USA), polyethylene terephthalate with a maximum particle size of 300 µm (Goodfellow, Cambridge, United Kingdom), polycarbonate beads sized 150–250 µm (Sigma- Aldrich, Saint Louis, MO, USA), polymethylmethacrylate analytical standard with a size of 50 μm in solution (Sigma-Aldrich, Saint Louis, MO, USA), polyamide (PA6, Nylon) with a mean size of 15–20 µm (Goodfellow, Cambridge, United Kingdom), polyurethane 3–5 mm (Goodfellow, Cambridge, United Kingdom), and the Polymer Kit 1.0 (Hawaii Pacific University, Honolulu, HI, USA). MAG water was obtained by filtering ultrapure water from a Arium® Mini water purification system connected to a CellPlus Ultrafilter (Sartorius, Göttingen, Germany) and three additional filtrations through a 5 μm polycarbonate filter thereafter.

To analyse microplastic content in commercially available tear-replacement solutions, two kinds of analysis were performed: MirrIR low-e microscope slides (Kevley Technologies, Chesterland, OH, USA) (slide) and on-filter analysis (filter). For every brand of eyedrop, two replicates of on-slide analysis and one on-filter analysis were conducted. For slide analysis, the entire content of a tear-replacement vial was filtered through a 5 μm pore size polycarbonate filter paper (Sartorius, Göttingen, Germany) mounted in a glass vacuum filtration unit with a funnel (Rocker, Kaohsiung, Taiwan) attached to a vacuum pump (Wiggens, Wuppertal, Germany). This filter was rinsed with 100 mL of MAG water. All particles were subsequently transferred onto a slide for analysis. On-filter analysis using a gold filter was further performed to avoid potential loss during transfer. Filtration was performed with 25 mm diameter, 0.8 µm pore size gold-coated polycarbonate membrane filters (Sterlitech, Aubrun, WA, USA). The entire content of one eyedrop vial was administered through the filter and washed with 100 mL of MAG water. Once dry, the filter was mounted on a filter holder (Agilent Technologies, Santa Clara, CA, USA) and analysed. For analysis of tear-replacement vial compositions, small fragments <300 µm of the vial was retrieved and analysed.

All samples were analysed to quantify polymer particles of sizes ranging between 20 and 500 µm on the Agilent 8700 LDIR chemical imaging system (Agilent Technologies, Santa Clara, CA, USA). Particles larger than 500 µm were not detected. Analyses were conducted via the automated Particle Analysis workflow as part of the Clarity software Version 1.5 (Agilent Technologies, Santa Clara, CA, USA). This automated workflow counts and sizes all particles.

An infrared spectrum is generated for particle identification (1800–975 cm^−1^) and the acquired spectra compared against an internal library. The library used in this study is based on the Systematic Identification of MicroPLastics in the Environment (siMPle), further modified for use with the LDIR chemical imaging system [20]. Additionally, information from open-source microplastics analysis repositories such as μATR-FTIR Spectral Libraries of Plastic Particles (FLOPP and FLOPP-e) were also integrated into the reference library [20, 21]. Finally, the library was further modified by Eurofins Environment Testing for the nine reported polymers which were verified against independently purchased reference materials.

Match quality was determined by comparing acquired spectra to the library reference spectra. A higher quality match score indicates a better fit between the measured spectrum and the material’s reference spectrum, with a score of 1 representing an absolute match. For this study, a minimum match quality above 0.7 was mandatory prior to inclusion in the count. This is in-line with reporting standards within the literature.

## Results

**Fig. 2:**
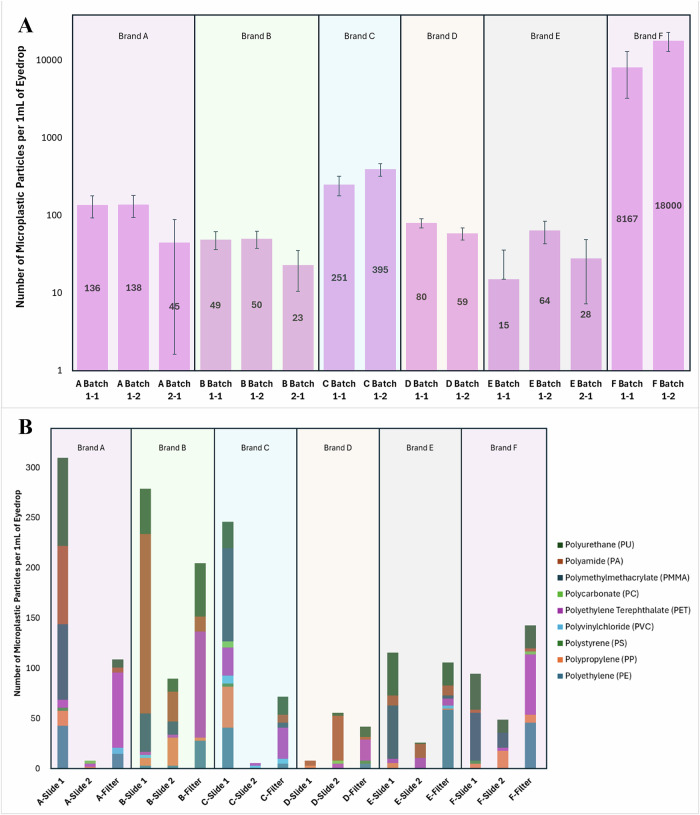
Results of light microscopy and laser direct infrared imaging system with spectroscopy analysis of Brands A-F. **A** Number of visually identified microplastic particles using light microscopy. Samples were obtained from across two production batches from each tear-replacement brand when possible. **B** Number and type of microplastic particles identified using laser direct infrared imaging system with spectroscopy. Two different kinds of analyses were used: Kevley slide analysis (slide) and on-filter analysis (filter).

The number of visually identified microplastic particles in each 1 mL sample via light microscopy is summarised in Fig. 2A with a logarithmic scale. Eyedrops from a separate production batch were not available for brands C, D, and F, hence light microscopy analysis was performed only for two samples from within the same production batch. Notably, Brand F had a cloudy appearance during analysis, and yielded the largest number of particles (>18,000 per mL of eyedrop) among all the tear-replacement samples tested. Even among eyedrops from the same production batch, the number of microplastic particles remained varied.

**Fig. 3:**
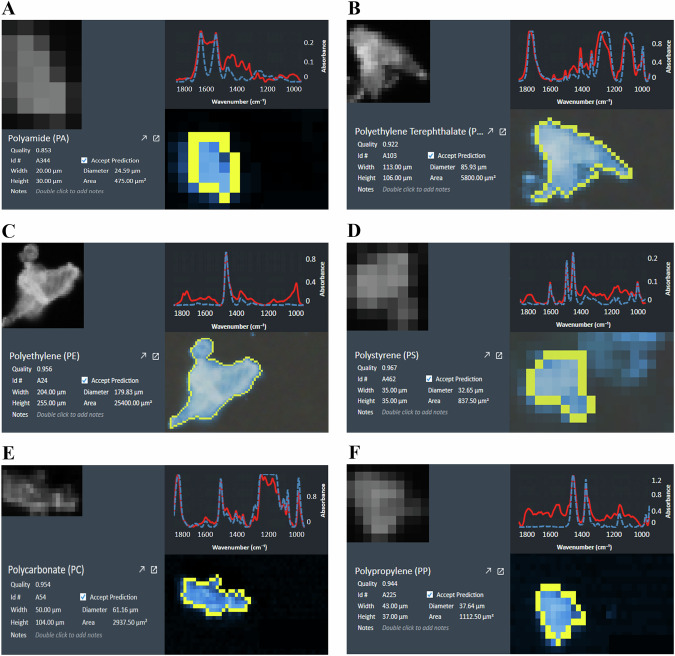
Representative images of microplastic particles identified in tear-replacement solutions using laser direct infrared imaging system with spectroscopy. **A** Polyamide. **B** Polyethylene terephthalate. **C** Polyethylene. **D** Polystyrene. **E** Polycarbonate. **F** Polypropylene. Blue dotted lines represent reference spectra and red solid lines represent measured spectra.

LDIR analysis on two replicates of on-slide analysis and one on-filter analysis were performed for all samples. All samples showed evidence of microplastic contamination (Table 2) (Fig. 2B). Nine polymer types were identified – polyamide, polycarbonate, polyethylene, polyethylene terephthalate, polymethylmethacrylate, polypropylene, polystyrene, polyurethane, and polyvinylchloride (Fig. 3). These microplastics were identified at varying levels in each tear-replacement sample and ranged between 20 and 500 μm in size. The amount and type of microplastic particles retrieved from each unit dose vial varied. This ranged between 5 and 308 particles per 1 mL of eyedrop. The number of microplastic particles varied even among samples from the same brand as evidenced by the high standard deviation (Table 2). Further analysis of tear-replacement solution vial materials confirmed a greater than 90% match of tested vials with polyethylene retrieved from tested samples of tear-replacement solutions.

**Table 2:** Number and type of microplastics per millilitre identified via laser direct infrared imaging system with spectroscopy.

Sample	Polyethylene (PE)	Polypropylene (PP)	Polystyrene (PS)	Polyvinylchloride (PVC)	Polyethylene Terephthalate (PET)	Polycarbonate (PC)	Polymethylmethacrylate (PMMA)	Polyamide (PA)	Polyurethane (PU)	Total	Mean number of microplastic particles (standard deviation)
A-Slide 1	43	15	3	0	8	0	75	78	88	308	141 ± 153
A-Slide 2	1	1	0	0	3	3	0	0	0	8	
A-Filter	15	0	0	6	75	0	0	5	8	108	
B-Slide 1	3	8	0	3	3	0	38	179	45	276	101 ± 151
B-Slide 2	3	28	0	0	3	0	13	30	13	88	
B-Filter	28	3	0	0	106	0	0	15	53	203	
C-Slide 1	41	41	3	8	28	6	93	0	26	241	106 ± 122
C-Slide 2	0	0	0	3	3	0	0	0	0	5	
C-Filter	5	0	0	5	31	0	5	8	18	71	
D-Slide 1	0	3	0	0	0	0	0	5	0	8	35 ± 24
D-Slide 2	0	0	0	0	5	3	0	45	3	55	
D-Filter	5	0	3	0	21	0	0	3	10	41	
E-Slide 1	0	6	0	0	4	0	53	10	43	115	82 ± 49
E-Slide 2	1	0	0	0	10	0	0	14	1	26	
E-Filter	59	1	0	3	7	0	3	10	23	106	
F-Slide 1	0	5	3	0	0	0	48	3	36	93	94 ± 47
F-Slide 2	0	18	0	0	3	0	15	0	13	48	
F-Filter	46	8	0	0	60	3	0	3	23	141	

## Discussion

### Variations in microplastics counts between samples of tear replacement solution

Our analysis revealed two distinct forms of variability: discrepancies between analytical methods employed, and variation in counts across different products and batches. Differences in quantification; with light microscopy identifying up to 18,000 particles in samples versus 35–141 particles by LDIR, underscores fundamental limitations of visual counting (Table 2, Fig. 2A) [19]. Visual counting has been reported to be susceptible to error rates of 20–70% and can be influenced by operator experience, sample complexity, and particle size [22, 23]. Our findings further demonstrate that without definitive chemical confirmation via spectroscopy, particle counts can be significantly overestimated.

For this definitive analysis, LDIR was chosen over other spectroscopic techniques, such as the Raman spectroscopy and scanning electron microscopy combination used by Choi et al. While Raman spectroscopy and scanning electron microscopy can identify smaller particles (as small as 200 nm), these methods are limited by higher costs and significantly longer analysis times [17, 24, 25]. Raman spectroscopy is further limited by fluorescence interference, with Choi et al. reporting exclusion of spectra with high fluorescence, which may have accounted for lower numbers of microplastics identified [17]. LDIR therefore affords reliable chemical identification for particles 20 µm and larger in a much shorter timeframe. However, LDIR has its own limitations; underestimation can occur if particles agglomerate, and its identification capability is dependent on the referenced spectral library [19]. To mitigate this, a highly robust database combining open-source and proprietary libraries and a strict spectral match quality above 0.7, consistent with regulatory standards was employed [20, 21, 26]. Beyond this methodological variance, variability observed within the LDIR results suggests the presence of complex and multi-faceted contamination issues within the product lifecycle itself.

Sources of contamination are likely numerous. While our analysis confirmed that the vials are likely a direct source of polyethylene particles, frequent detection of other polymers such as polyamide and polyurethane suggests that vial degradation is not the only cause. We postulate that these additional contaminants could have been introduced at various points along the supply chain. This includes sources such as raw chemical ingredients or shedding from equipment during the manufacturing process. Furthermore, post-manufacturing conditions such as physical stress during transport, temperature fluctuations in storage, and even the shearing force applied while twisting the cap can influence plastic degradation and release microplastic particles, contributing to the variance observed [27, 28].

### Limitations and future directions

We acknowledge several limitations to this study. The lower detection limit of LDIR (20 µm) means smaller, potentially more hazardous, nano- and microplastic particles were not quantified in this study. Additionally, this pilot study, by design, analysed a small number of samples per brand to allocate limited resources towards surveying a wider range of products.

Given the significant implications of these preliminary findings, all LDIR analyses were conducted in a National Association of Testing Authorities (NATA) accredited facility by experienced experts to ensure the accuracy and rigor of presented results. Ultimately, this variability, while analytically challenging, is likely reflective of the true, real-world conditions these products endure from production to the consumer.

### Clinical and regulatory implications

This investigation illuminates a clinical paradox: the very therapeutics prescribed to treat ocular ailments may expose an already compromised ocular surface to a significant burden of microplastic contaminants. Addressing this challenge requires concerted, multi-stakeholder efforts from scientists, pharmaceutical manufacturers and regulatory authorities to systematically identify and eliminate sources of contamination. Furthermore, urgent research is needed to develop and validate low-shedding primary packaging, addressing the critical knowledge deficit in particulate generation from these materials. Ultimately, the implementation of rigorous, industry-wide regulations and standardised quality control testing for microplastics in all ophthalmic therapeutics is crucial to safeguard patients and ensure the integrity of manufactured products.

## Conclusion

The discovery of microplastics in ocular therapeutics raises significant concerns for human health, and represents a rapidly developing field of study. Urgent research is required to identify the presence, origin, and characteristics of these contaminants, and to determine their full impact on ocular and systemic well-being. This foundational work is essential to proactively minimise patient exposure and mitigate potential harm.

## Summary

### What was known before

- Microplastics are ubiquitous and are found in most healthcare environments.
- Microplastics have the potential to cause adverse ophthalmic effects on the ocular surface.

### What this study adds

- Microplastics have been identified in commonly used over-the-counter tear-replacement solutions.
- Further studies are urgently required to characterise the impact of microplastics in ophthalmic therapeutics on ocular and systemic health.
- To ensure patient safety and product integrity, implementation of stringent, industry-wide regulations and standardised testing protocols for microplastics in all ophthalmic therapeutics is recommended.

## Data Availability

Data analysed during this study are included in this published article. Specific information is available from the corresponding author on reasonable request.
